# *Exiguobacterium sp.* A1b/GX59 isolated from a patient with community-acquired pneumonia and bacteremia: genomic characterization and literature review

**DOI:** 10.1186/s12879-017-2616-1

**Published:** 2017-07-21

**Authors:** Xingchun Chen, Lijun Wang, Jiali Zhou, Honglong Wu, Dong Li, Yanchao Cui, Binghuai Lu

**Affiliations:** 1 Department of Laboratory Medicine, People’s Hospital of Guangxi Zhuang Autonomous Region, Nanning, 530021 China; 2 Department of Laboratory Medicine, Beijing Tsinghua Chang Gung Hospital, Tsinghua University, Beijing, 102218 China; 3 BGI Tianjin, Tianjin, 300308 China; 4 Department of Laboratory Medicine, Civil Aviation General Hospital, Peking University Civil Aviation School of Clinical Medicine, No1. Gaojing Street, Chaoyang District, Beijing, 100123 China

**Keywords:** *Exiguobacterium* spp., Whole genome sequencing, Virulence factors, Antibiotics, Bacteremia, Community-acquired pneumonia

## Abstract

**Background:**

Bacterial species belonging to the genus *Exiguobacterium* are facultative anaerobic, non-spore-forming, Gram-positive bacilli, and rarely associated with human infections. Herein, we reported the first case of community-acquired pneumonia (CAP) and bacteremia due to *Exiguobacterium* spp. in China.

**Case presentation:**

An adult male with severe CAP was hospitalized. The pathogen was isolated from his bloodstream and broncho-alveolar lavage fluid. The correct identification of the micro-organism was achieved using 16S rRNA sequencing, and its antibiotic susceptibility test was performed by microdilution method. The Whole Genome Sequencing (WGS) was used to characterize its genetic features and to elucidate its potential pathogenic mechanisms. Furthermore, its genome sequence was also compared with those of 3 publicly-available *Exiguobacterium* strains. A PubMed search was performed for further understanding the features of *Exiguobacterium* infections. Phylogenetic analysis of the 16S rRNA gene sequence showed that the strain GX59 was most closely related to *Exiguobacterium* AT1b (99.7%). The genome of GX59 was 2,727,929 bp in size, harbouring 2855 putative protein-coding genes, 5 rRNA operons, 37 tRNA genes and 1 tmRNA. The multiple genome comparison of 4 *Exiguobacterium* strains demonstrated that *Exiguobacterium* contained 37 genes of secretion systems, including *sec*, *tat*, *FEA*, Type IV Pili and competence-related DNA transformation transporter (*Com*). Virulence factors of the micro-organism included *tlyC, NprR, MCP, D*am, which might play a critical role in causing lethal infection.

**Conclusions:**

The study highlighted the potential pathogenicity of the genus *Exiguobacterium* for its unique genes encoding various virulence factors and those associated with antibiotic resistance, therefore, its clinical significance should be valued.

## Background

The genus *Exiguobacterium* belongs to the group of *coryneform* bacteria, firstly described in 1983 by *Collins* et al. [1]. It is a facultative anaerobic, Gram-positive bacillus. The pathogenic potential of *Exiguobacterium* spp. seems rather low. To date, only a few cases of bacteremia and skin infection were documented in English literature [2–6]. However, the clinical infection due to the micro-organism might be probably underdiagnosed or unreported, for it tended to be misidentified by routine commercial methods [2–6]. As an emerging pathogen, its pathogenesis should be clarified.

Here, we present a case of community-acquired pneumonia (CAP) and bacteremia due to *Exiguobacterium* sp. strain AT1b/GX59 in a type 2 diabetes mellitus (T2DM) patient. To the best of our knowledge, this is the first fatal case of CAP and bacteremia due to the micro-organism in a healthy male without serious underlying diseases. In order to generate significant insights into pathogenicity of the micro-organism, its genome was sequenced and compared with 3 *Exiguobacterium* genomes available in NCBI Genbank database.

## Case presentation

### Medical history

On July 28th, 2014, a 51-year-old male with severe pneumonia, acute respiratory distress syndrome was admitted to People’s Hospital of Guangxi Zhuang Autonomous Region at Guangxi, China. He is a farmer living on planting sugarcane and had a history of 8-year T2DM.

At admission, he was febrile (37.8 °C), with a respiratory rate of 60 breaths/min, a pulse rate of 126 beats/min and blood pressure of 165/80 mmHg. He also complained chill, headache, cough, hemoptysis and dyspnea for one day. Physical examination showed that he was in respiratory distress due to chest pain and tightness. Chest CT scan indicated diffuse pulmonary lesions and consolidation. His peripheral leukocyte count was 1.54 × 10^9^/L with 53.9% neutrophils. His blood glucose was badly controlled at 18.0 mmol/L. Results of initial arterial blood gas analyses were: pH 7.42, carbon dioxide pressure (PCO_2_) 27 mmHg, and oxygen pressure (PO_2_) 31 mmHg. His liver function tests were within reference ranges; however, his creatinine was 183 μmol/L (reference range 54–106 μmol/L) and urea was 12.7 mmol/L (3.2–7.1 mmol/L). Afterwards, broncho-alveolar lavage fluid (BALF) and 3 sets of blood sample were collected for culture. Furthermore, imipenem combined with voriconazol was empirically administered in an attempt to relieve infection.

Two days after our empirical therapy, his blood pressure dropped to 108/55 mmHg, and arterial blood gas analysis showed: pH 7.26, PCO_2_ 55 mmHg, and PO_2_ 53 mmHg. The marked changes in lung stroma were detected on CT screening. Poor lung compliance, and following alveolar rupture and subcutaneous emphysema brought the patient into a critical condition. On August 1st, the patient fell into a coma and was brought back home by his family members. He died two days later.

### Microbiologic test

**Fig. 1:**
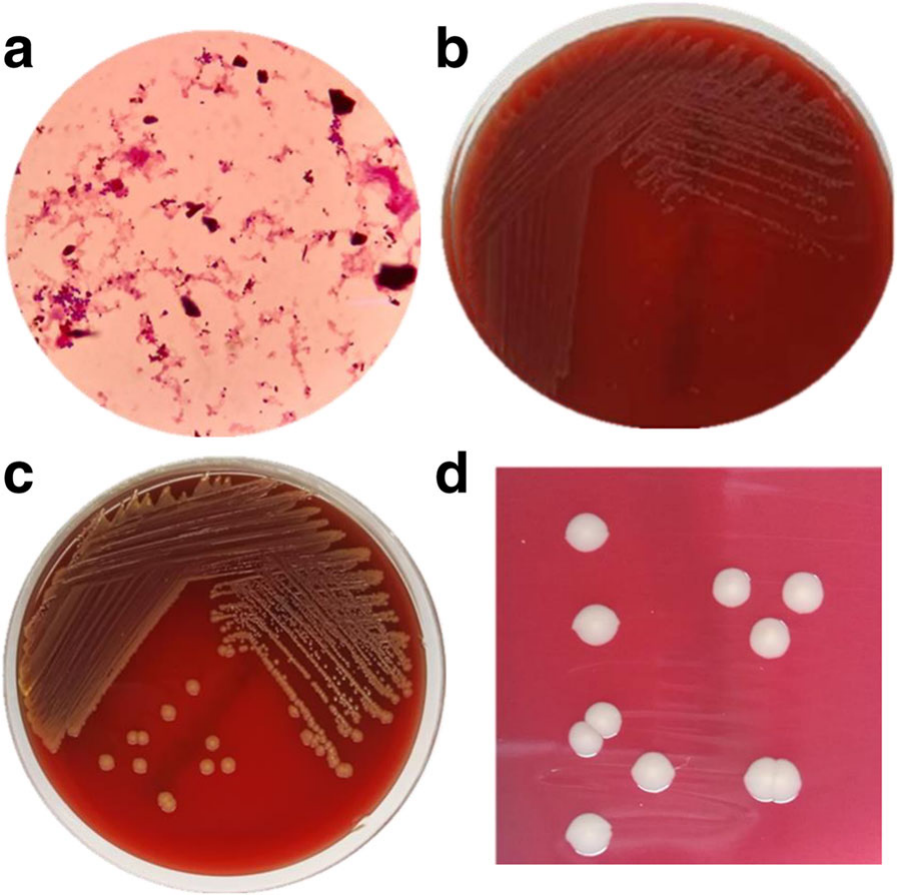
Appearances after 24 h and 48 h incubation on Blood agar plates. **a** Gram staining of the positive blood bottle; **b** *Exiguobacterium* AT1b/GX59 after 24 h growth; **c** and **d** after 48 h growth

Microbial growth was detected in 3 aerobic blood culture bottles obtained through separate needle puncture sites. Direct gram-stain demonstrated Gram-positive, short, and straight rods. Positive blood culture broths were subcultured at 37 °C under 5.0% CO_2_. The non-hemolytic, gray colonies were observed on blood agar from both blood and BALF cultures, but turned yellow after 48 h, and there was no growth on MacConkey agar, as shown in Fig. 1. The isolate was catalase positive and oxidase negative. Phenotypic identification of the bacteria by the ANC (BioMerieux, France) card yielded poor identification of *unidentified organism* (isolate Bionumber: 6,521,100,600,035). Antimicrobial susceptibility testing was performed by using micro dilution and E-test method. The MIC results were as follows: susceptible to penicillin (0.064 μg/ml), meropenem (0.064 μg/ml), gentamicin (0.25 μg/ml), ciprofloxacin (0.25 μg/ml), rifampin (0.125 μg/ml), and vancomycin (0.125 μg/ml), but resistant to tetracycline (16 μg/ml), erythromycin (4 μg/ml) and clindamycin (1 μg/ml).

### 16S rRNA sequencing and phylogenetic analyses

**Fig. 2:**
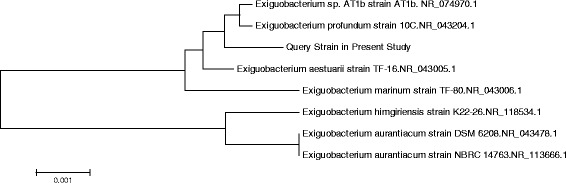
Phylogenetic relatedness of query isolate to closest neighbors based on 16S rRNA homology. Neighbor-joining tree based on 16S rRNA (1,443bases) sequences, showing the phylogenetic relationship between query strain in present study and other related members of the genus *Exiguobacterium*

The 16S rRNA sequencing was conducted to identify the pathogen. Other 7 related members of the genus *Exiguobacterium* available in NCBI Genebank database were included for phylogenetic analyses. The sequence analysis of a 1433 bp segment of the 16S rRNA genes of the organism demonstrated an identity of 99.7% with *Exiguobacterium. AT1b* (GenBank accession no. NR_074970.1) (Fig. 2).

### Whole genome sequencing (WGS)

To elucidate the potential pathogenicity of the micro-organism in current study, we sequenced its whole genome using a whole-genome shotgun strategy based on the Illumina HiSeq platform. The high-quality reads were generated after filtering low-quality ones, adapter contamination and PCR primers by using the software Trimmomatic (version 0.32). De novo assembly was performed with SOAP denovo (version 2.0.1), an empirically-improved memory-efficient short-read de novo assembler, and gaps were closed by Gap Closer (version 1.12). Furthermore, Gene prediction and annotation were carried out by PROKKA pipeline for rapid prokaryotic genome annotation.

This whole-genome shotgun sequencing project has been deposited at DDBJ/ENA/GenBank under the accession GenBank accession no. GCA_001908175.1. WGS generated 6,916,887 bp pair-end reads with read length of 150 bp. A total of 21 scaffolds were assembled, with an N50 size of 539,676 bp, an N90 size of 103,057 bp and the largest scaffold size of 632,933 bp. The GC content of GX59 was 47.49%, similar to that of other *Exiguobacterium* species. The size of GX59 genome was 2,727,929 bp, containing 2855 putative protein-coding genes, 5 rRNA operons, 37 tRNA genes and 1 tmRNA. The KEGG annotation of the GX59 genome was performed by BlastKOALA, and 52.9% of 2812 entries were annotated (Table 1). The majority of metabolism genes in the genus *Exiguobacterium* enabled it to survive in various environments. BRITE reconstruction result demonstrated that GX59 strain contained 13 antimicrobial resistance genes, including tetracycline resistance genes (*tetB*, and efflux pump *Tet38*), macrolide resistance genes (*msrA, vmlR, mef,* and *vat*), aminoglycoside resistance genes (*aacC, aac6-I, aacA7, aadA,* and *aadK*), phenicol resistance genes (*catA*), cationic antimicrobial peptide (CAMP) resistance genes (*mprF* and *fmtC*), multidrug resistance efflux pump (*abcA* and *bmrA*), and vancomycin resistance modules (*vanY* and *vanW*).

**Table 1:** Summary of *Exiguobacterium* AT1b/GX59 strain KEGG annotation

KEGG (functional category)	GX59 (n)
Genetic Information Processing	418
Environmental Information Processing	351
Amino acid metabolism	161
Carbohydrate metabolism	161
Cellular Processes	172
Metabolism of cofactors and vitamins	103
Nucleotide metabolism	88
Energy metabolism	80
Human Diseases	75
Enzyme families	58
Lipid metabolism	47
Metabolism of other amino acids	35
Metabolism of terpenoids and polyketides	32
Glycan biosynthesis and metabolism	30
Organismal systems	31
Xenobiotics biodegradation and metabolism	25
Biosynthesis of other secondary metabolites	17
Unclassified	231

### Ortholog analysis

The genome sequences of *Exiguobacterium* sp. AT1b (GenBank accession no. NR_074970.1), *E. aurantiacum* DSM 6208 (GenBank accession no. NZ_JNIQ01000001.1), and *E. acetylicum* DSM 20416 (GenBank accession no. NZ_JNIR01000001.1) were collected from NCBI Nucleotide database. The protein sequences of above genomes and that of AT1b/GX59 in current study were collected together and searched against itself via multiparanoid program, based on the Blastp algorithm with the following criteria: identity ≥ 50%, coverage ≥ 50%, BLAST score ≥ 50, and confidence score = 1. Afterwards, unique genes of AT1b/GX59 and those shared by all 4 strains were parsed out from the blast results and annotated by KEGG orthology (KO) identifiers in the web-based server called KAAS (KEGG Automatic Annotation Server: http://www.genome.jp/kegg/kaas/).

### Virulence factors related to pathogenicity in *Exiguobacterium* strain GX59 by WGS

**Fig. 3:**
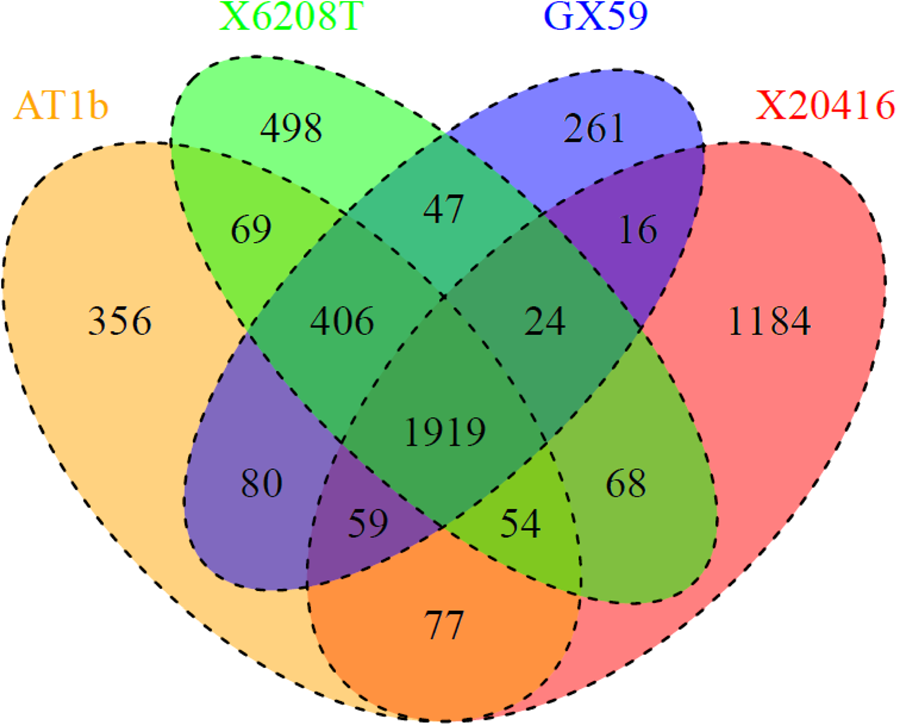
Venn diagrams of common- and specific-gene in *E. acetylicum* DSM 20416, *E. aurantiacum* DSM 6208, *Exiguobacterium* sp. AT1b and *Exiguobacterium* AT1b/GX59 strain in current study

A total of 261 specific genes of *Exiguobacterium sp.* A1b/GX59 were identified. Moreover, the comparison of general genome features within the 4 *Exiguobacterium* strains indicated that they shared 1919 core genes, which participated in metabolic, cellular, and genetic information processing, respectively (Fig. 3 and Table 2). Of the 1919 shared genes, 65.5% were annotated with KEGG. A variety of secretion system genes involved in pathogenicity mechanism were also identified. The 4 *Exiguobacterium* strains possessed 37 genes of secretion systems, encoding two translocons, including *sec* (secretion), *tat* (twin-arginine translocation), *FEA* (flagella export apparatus), *FPE* (fimbrilin-protein exporter), Type IV Pili and competence-related DNA transformation transporter (*com*).

**Table 2:** General genome features of the four *Exiguobacterium* species

Organism	Source	Size(Mb)	GC%	CDS	No. of tRNAs	No. of contigs	Genome status	GenBank No.
*Exiguobacterium* AT1b/GX59 strain	BALF and blood, China	2.73	47	2855	37	21	Draft	GCA_001908175.1
*Exiguobacterium* sp. AT1b	spring water, USA	2.99	48	3020	68	/	Complete	CP001615
*E. acetylicum* DSM 20416	Creamery waste, UK	3.28	47	3323	69	3	Draft	JNIR00000000
*E. aurantiacum* DSM 6208	Potato wash, UK	3.04	53	3067	67	2	Draft	JNIQ00000000

Furthermore, 261 specific genes of *Exiguobacterium sp.* A1b/GX59 were submitted in KAAS, and 24.9% were annotated as hypothetical proteins with the BRITE functional hierarchy. In its genome, a series of unique virulence genes were identified, including *tlyC* (AT1b/GX59-P-00125 k03699) encoding hemolysin, a type of membrane-damaging toxin, *NprR* (AT1b/GX59-P-01697 k20480) encoding a quorum-sensing receptor, *mcp* (methyl accepting chemotaxis proteins) (AT1b/GX59-P-01386 k03406) and *Dam* (DNA adenine methylase) (AT1b/GX59-P-01413 k06223). Moreover, in secretion systems, *Exiguobacterium sp.* A1b/GX59 encompassed an extra gene *SecDF*, which played a role as a chaperone facilitating the translocation of *L. monocytogenes* virulence factors during infection [7].

### Literature review

To better understand the characteristics of *Exiguobacterium* infections, PubMed was searched and 5 related reports were included for comparison [2–6].

## Discussion

The clinical characterizations of *Exiguobacterium* species isolated previously from different infectious samples were listed in Table 3 [2–6]. As documented, most infections due to *Exiguobacterium* spp. had underlying diseases, such as liver cirrhosis [2], intravenous drug abuse and multiple myeloma [6]. However, the patient in present study was in a generally healthy condition, though he suffered from T2DM. Although a male patient infected by the micro-organism was previously healthy, different from our patient, he had only an ulcer on a finger with a painful black eschar rather than systematic infection [4]. Bacteria of the genus *Exiguobacterium* distribute extensively and have been isolated from markedly diverse sources, including water, the rhizosphere of plants, and the environment of food processing plants [8]. The patient in current study was a farmer living on processing plants in humid climate of South China. Considering his early clinical symptoms, inhalation of the micro-organism might be a possible portal of entry of his pneumonia [8].

**Table 3:** Summary of reported cases of human infection due to *Exiguobacterium spp.*

Reference	2003 [2]	2006 [5]	2007 [3]	2014 [4]	2007 [6]	Our data
Cases number	1	1	1	1	6	1
Age(y)/Gender	55/M	NM/M	92/F	66/M	27/M(1), NM/M(3),neonate(1),55/M(1)	51/M
Community-acquired or hospital acquired	NM	NM	Nosocomial acquisition	Community-acquired	NM	Community-acquired
Sources	NM	NM	Catheter-related	Handled the skin of a deer and a wild boar.	NM	Respiratory tract, sugarcane farmer
Underlying disease	Alcoholic liver cirrhosis.	NM	Hypertension, hyperuricaemia and Alzheimer’s disease.	Previously healthy	Intravenous drug abuse(1), multiple myeloma(2), suspected infective endocarditis(1), neonate(1), igg kappa multiple myeloma, received local radiotherapy, corticosteroids and infusion chemotherapy(1).	T2DM
Presentation	Abdominal pain and diarrhea	NM	37.4 °C; late increased to 38.6 °C.	Afebrile with no systemic symptoms. Ulcer on a finger with a painful black eschar.	NM(5), febrile at 38.2 °C and experienced rigors after the indwelling central line was flushed(1).	Fever, chills, headache, cough, expectoration, hemoptysis, and dyspnea
Diagnosis	Bacteremia	Bacteremia	Bacteremia	Cutaneous infection	Bacteremia	Bacteremia and pneumonia
Sources	Bloodstream	Bloodstream	Bloodstream	Skin infection exudate	Bloodstream	Bloodstream and BALF
Identification						
Commercial identification systems	*Pantoea agglomerans* by Enterotube II (Becton Dickinson Diagnostic Systems, Sparks, MD, USA) and Phoenix Identification System PMIC/ID-30 (Becton Dickinson Diagnostic Systems)	*Oerskovia xanthincolytica* by API Coryne (biomérieux)	Not identified by API Coryne (biomérieux)	*Bacillus spp.*	*Cellulomonas/Microbacterium spp.* By API Coryne (biomérieux)	*Unidentified organism* by ANC card (biomérieux)
16 s rRNA	99% *E. profundum* (hm584043.1)	*Exiguobacterium sp.* 99% identity of 1024 nucleotides.	*E. acetylicum* (99% identity of 506 nucleotides)	*E. sibiricum* (1413 bp, and similarity was 99.6%)	*E. aurantiacum* (high sequence homology, 99.2%)	*Exiguobacteriu* sp. *At1b*. (1433 bp and similarity 99.7%)
AST	NM	NM	Susceptible to penicillins, cephalosporins, Aminoglycosides and quinolones	Susceptible to penicillin, cefotaxime, imipenem, levofloxacin, vancomycin, clindamycin, erythromycin, gentamicin, doxycycline, linezolid, and daptomycin.	Susceptible to ampicillin, cefotaxime, chloramphenicol, ciprofloxacin, clindamycin, erythromycin, gentamicin, penicillin, rifampicin, teicoplanin, tetracycline and trimethoprim	Susceptible to penicillin, meropenem, gentamicin, ciprofloxacin, rifampin, and vancomycin, Resistant to tetracycline, erythromycin, clindamycin
Antibiotic therapy	NM	NM	Intravenous cefuroxime treatment was initiated; afterwards, cefuroxime	Ciprofloxacin for 10 days.	6th patient: intravenous ceftazidime and teicoplanin for the following 3 days, the fever persisted. Others: unknown	Imipenem, moxifloxacin and voriconazole
Outcome	Recovered	NM	Recovered	Recovered	Recovered	Died

*F* Female, *M* Male, *NM* Not mentioned, *AST* Antimicrobial Susceptibility Testing

The literature review demonstrated that *Exiguobacterium* were rarely isolated as human pathogen. Furthermore, it is difficult to identify *Exiguobacterium* spp. based on traditional biochemical method. Almost all reported infective strains of this genus were misidentified when the commercial biochemical system was used, including API Coryne kit/VITEK 2 Compact system (Bio-Mérieux, France), and Becton Dickinson Diagnostic Systems [2–6]. Furthermore, Matrix-assisted laser desorption ionization–time of flight (MALDI-TOF) analysis is presently becoming a routinely used tool in many microbiology laboratories, and might be used in identifying the genus *Exiguobacterium* in future [6], however, in our study, the strain GX59 was identified as *Exiguobacterium aurantiacum* 290RLT with its score as 1.547 by using MALDI-TOF (Bruker Daltonics, MALDI Biotyper 3.1 software, 2371 species and 5989 entries included).

In current study, the pathogen could not be identified using ANC card of VITEK 2 system, and was confirmed as *Exiguobacterium* AT1b by 16s rRNA sequencing only in retrospective analysis. *Exiguobacterium* AT1b was initially isolated from a slightly alkaline, highly carbonate, and hot spring water sample from Yellowstone National Park [9], and had never documented to be isolated clinically. Therefore, the in-depth analysis of the *Exiguobacterium* sp. AT1b/GX59 isolate in present study might elucidate the pathogenicity of this environmental saprophytic micro-organism. Moreover, the comparison study of the genomes of the strain GX59 and other *Exiguobacterium* spp. might then shed light on the pathogenicity of the microorganism. The publicly-available genomes of *Exiguobacterium* spp. were mostly isolated from environment. Through ortholog gene analysis, it was reasonably to speculate that several following genetic characteristics were closely related to the pathogenicity of current GX59 strain. Firstly, secretion systems identified in the strain might be involved its pathogenicity. Desvaux M [10] suggested to use the terms *sec*, *tat*, *FEA*, and *FPE* in translocation systems across the cytoplasmic membrane of both Gram-positive and -negative bacteria. The above 4 *Exiguobacterium* strains possessed 37 genes of secretion systems, encoding two translocons, including *sec, tat, FEA*, and competence-related DNA transformation transporter (*Com*). In pathogenic Gram-positive bacteria, the vast majority of proteins were exported out of cytosol by conserved general *sec* system [11] or, by *tat* system [12]. Other studies discovered *Com* proteins were required for internalization of extracellular DNA [13, 14]. Taken together, *Exiguobacterium* had the ability to export toxins to exert its full virulence and to uptake DNA to acquire a variety of virulence and resistance traits. Secondly, a series of unique virulence genes identified in the GX59 strain genome might explain its high virulence. For example, the hemolysin encoded by the gene *tlyC* was taken as a virulence factor in a variety of Gram-positive infectious bacteria. Carvalho E [15] suggested that the protein *tlyC* was not directly involved in hemolysis, but contributed to binding of Leptospira to extracellular matrix (ECM) during host infection. The non-hemolytic GX59 strain in present study contained *tlyC* genes, in consistent with Carvalho E’s finding [15]. Moreover, NprR, as a major transcriptional regulator, was documented to belong to RNPP family of quorum-sensing (QS) receptors, a group of intracellular regulators activated directly by signaling oligopeptides in Gram-positive bacteria [16]. It might control sporulation and necrotrophic properties, ensuring survival and dissemination of the bacteria in clinical infections by feeding on host proteins [16]. Another pathogenetic gene uniquely detected in the GX59 strain was *mcp*, which got involved in virulence, motility, and biofilm formation of bacteria [17], possibly performing a potential function in invasive infections. Furthermore, *Dam* mediated the methylation of adenine in the 5′-GATC-3′ sequence shortly after DNA replication, and was implicated as a virulence factor in bacterial pathogenesis. As documented previously, *Dam* methylation was required for efficient biofilm production in *Salmonella enterica* serovar enteritidis [18]. *Dam* was also crucial in modulating the pathogenicity of *K. pneumoniae* genotype K1 [19]. The *Dam* gene identified in the GX59 strain might participate in its invasiveness during infection. Finally, apart from the virulence factors unique in the AT1b/GX59 strain, other common virulence factors might be involved in severe infection. For, example, the GX59 strain harboured an extra gene *SecDF*, which played a role as a chaperone that facilitates the translocation of *L. monocytogenes* virulence factors during infection [7]. Deletion of *secDF* resulted in reduced virulence and motility *Bacillus cereus* ATCC 14579 [20]. Taken together, the severe CAP and following bacteriemia in our patient was possibly explained by the high pathogenicity due to the above-identified virulence genes.

Except for high pathogenic genes, *Exiguobacterium* spp. also harboured some antimicrobial resistance genes, including tetracycline resistance genes, macrolide resistance genes, aminoglycoside resistance genes, phenicol resistance genes, cationic antimicrobial peptide, multidrug resistance efflux pumps (*abcA* and *bmrA*), and vancomycin resistance modules (*vanY, vanW*). Accordingly, although the strain remained susceptible, it would easily become resistant to many antibiotics. Generally, timely antibiotic therapy often resulted in a favorable outcome in the clinical infections due to the microorganism [2, 3, 5, 6]. However, in current study the isolate was susceptible to the antibiotics used, e.g. meropenem and ciprofloxacin, but the patient died of deteriorated infection. This might be explained by the reasons as follows. The pathogen was not identified timely, the appropriate antibiotic therapy failed to take, and furthermore, the rapidly-deteriorated severe acute type 2 respiratory failure caused his death.

## Conclusions

In summary, the *Exiguobacterium* sp. AT1b/GX59 strain in current study is equipped with a variety of factors that facilitate its adaptation to a pathogenic lifestyle, such as hemolysin, secretion systems, chemotaxis proteins, and antibiotic resistance genes. The *Exiguobacterium* sp., as a potential pathogen, should attract more attention.

## Abbreviations

BALF: broncho-alveolar lavage fluid
CAP: community-acquired pneumonia
T2DM: type 2 diabetes mellitus
WGS: Whole Genome Sequencing
